# Prevalence and psychiatric comorbidities of night-eating behavior in obese bariatric patients: preliminary evidence for a connection between night-eating and bipolar spectrum disorders

**DOI:** 10.1007/s40519-021-01306-1

**Published:** 2021-10-06

**Authors:** Giulio Emilio Brancati, Margherita Barbuti, Alba Calderone, Paola Fierabracci, Guido Salvetti, Francesco Weiss, Ferruccio Santini, Giulio Perugi

**Affiliations:** 1 Psychiatry 2 Unit, Department of Clinical and Experimental Medicine, University of Pisa, Via Savi 10, 56126 Pisa, Italia; 2 Endocrinology Unit, Department of Clinical and Experimental Medicine, Obesity and Lipodystrophy Research Center, University Hospital of Pisa, Pisa, Italy

**Keywords:** Night-eating, Mood disorder, Bipolar disorder, Obesity, Bariatric surgery

## Abstract

**Purpose:**

The co-occurrence of obesity, eating and mood disorders has been frequently reported in clinical and epidemiological settings. This study aimed to explore the prevalence of night-eating obese patients referred for bariatric surgery and to identify associated psychopathology and psychiatric comorbidity.

**Methods:**

The sample was composed of 121 obese patients consecutively enrolled between November 2010 and May 2012 during psychiatric evaluations for bariatric intervention. Clinical features and psychiatric diagnoses were collected. Night-eating was investigated through the administration of the Night-eating Questionnaires (NEQ) and was defined as the presence of self-reported evening hyperphagia and/or nocturnal ingestions. Binge-eating and purging behaviors and general psychopathology were respectively assessed using the Bulimic Investigatory Test, Edinburgh and the Symptom Checklist-90-Revised.

**Results:**

Night-eating was reported by twenty subjects (16.5%). Patients with night-eating behavior were significantly more frequently diagnosed with bipolar spectrum disorders and with comorbid eating and mood disorders in comparison with other patients. Night-eating patients showed significantly more binging/purging behaviors and greater severity of somatization, obsessive–compulsive symptoms, phobic anxiety, psychoticism and sleep disorders. Patients with bipolar disorder type 1 or 2 scored significantly higher than those without mood disorders at NEQ total score, mood/sleep and nocturnal ingestions subscales, but also scored significantly higher than other patients with mood disorders at the latter subscale.

**Conclusion:**

Patients with evening hyperphagia and/or nocturnal ingestions should be carefully evaluated to detect possible bipolar spectrum disorders and other eating disorders. Prompt management of these conditions should be provided before bariatric interventions.

**Level of evidence:**

V, cross-sectional descriptive study.

**Supplementary Information:**

The online version contains supplementary material available at 10.1007/s40519-021-01306-1.

## Introduction

Night-eating syndrome was first described by Stunkard et al. [1] in a small sample of morbidly obese patients. In this early description, it was depicted as an eating disorder characterized by three core symptoms: morning anorexia, evening hyperphagia (the consumption of more than 25% of daily calories after the evening meal), and sleep-onset insomnia. Over the years, several changes to these diagnostic criteria have been proposed and the lack of an internationally shared definition over the years has contributed to considerable variation across studies in its prevalence and clinical characterization. Importantly, the criterion regarding nocturnal ingestions was added [2], whereas morning anorexia was removed from core symptoms required for the diagnosis and included among additional features characterizing the disorder [3].

Recently, night-eating syndrome was included in the 5th edition of the Diagnostic and Statistical Manual of Mental Disorders (DSM-5) [4] among “other specified feeding or eating disorders”, giving way to its possible recognition as an independent diagnostic category. According to DSM-5, night-eating syndrome is defined as “recurrent episodes of night-eating, as manifested by eating after awakening from sleep or by excessive food consumption after the evening meal”. Awareness and recall of the eating should be present and significant distress and/or functional impairment are required. Frequency and duration criteria were not specified.

Despite this progress, night-eating is still poorly described and under-investigated. The relationship between night-eating and obesity is still controversial and clinical research has only recently begun to examine the possibility that night-eating may contribute to obesity as well as binge-eating [5, 6]. While night-eating has been found to rarely occur in the general population (1.1–1.5%) [7], it is relatively common in obese patients (6–16%) [8], especially in those undergoing bariatric surgery (2–55%) [7]. In addition, the high prevalence of obesity/overweight in patients with night-eating seems to confirm this association [9]. Night-eating also shows important, but yet to be elucidated, relationships with various psychiatric disorders, often found in comorbidity, such as mood, eating, anxiety, sleep and substance use disorders [10–12].

Circadian mood disturbances seem to represent an important clinical aspect of night-eating, and depressed mood in the evening has been proposed among optional criteria for the diagnosis of night-eating syndrome [2]. In recent years, there has been increasing evidence pointing to night-eating as an eating disorder characterized by a desynchronization between the circadian rhythms of eating and sleep, resulting in a phase delay of food intake [3, 7]. Indeed, significant changes in timing and amplitude of neuroendocrine circadian markers involved in appetite and sleep regulation, such as leptin, insulin and melatonin, have been observed in patients with night-eating syndrome [13].

Importantly, circadian rhythms disruption has also been implicated in the pathogenesis of mood disorders [14] and is increasingly considered a core feature of bipolar disorder (BD) [15].

Sleep disturbances, delayed sleep–wake rhythms disorders and abnormalities in melatonin secretion have been repeatedly observed in euthymic patients with BD [15]. Moreover, BD patients often show an evening chronotype that is associated with disorganized eating behavior, unhealthy food preferences and nocturnal binges [16]. Accordingly, a strong relationship between night-eating and BD could be hypothesized. Nevertheless, only one study specifically examined the prevalence and correlates of night-eating in patients with BD [17]. Night-eating was found to commonly occur in a sample of 80 euthymic BD patients, with about a 10% prevalence, and was significantly associated with anxiety, worse functioning, evening preference and sleep disorders [17]. Despite the relatively high prevalence of bipolarity in pre-bariatric subjects [18–20], the association between night-eating and BD in morbidly obese patients has not been investigated so far.

Our study aimed to examine the prevalence of night-eating behaviors in a population of obese patients referred for bariatric surgery. Our second objective was to describe psychiatric comorbidity and psychopathology characterizing night-eating patients, comparing these latter with obese patients without night-eating. We hypothesized that night-eating might show strong associations with psychiatric comorbidity, especially with mood disorders belonging to the bipolar spectrum.

## Patients and methods

The study sample was composed of 121 obese patients (BMI ≥ 30 kg/m^2^) referred for bariatric surgery to the Obesity Center of the Endocrinology Unit in Pisa University Hospital. Patients were consecutively recruited between November 2010 and May 2012. All patients were adult (≥ 18 years) and provided written informed consent to data collection for research purposes. During presurgical evaluation, all patients were routinely interviewed by licensed psychiatrists. In a single consultation, current and lifetime psychiatric diagnoses were assessed by using the Structured Clinical Interview for DSM-IV Axis I disorders [21]. Major depressive disorder, bipolar spectrum disorders (i.e., BD type 1 and 2, and cyclothymia/other specified bipolar disorder), panic disorder, and eating disorders (i.e., anorexia and bulimia nervosa, and binge eating disorder) were specifically investigated.

Current eating disorders and general psychopathology were also investigated using a series of validated self-report questionnaires, namely the Night-eating Questionnaire (NEQ) [22, 23], the Bulimic Investigatory Test, Edinburgh (BITE) [24], and the Symptom Checklist-90-Revised (SCL-90-R) [25].

Night-eating was investigated using the Italian version of the NEQ, a self-rated instrument commonly used for the screening of night-eating syndrome [22, 23]. The questionnaire consists of 14 items subdivided into four facets, namely morning anorexia, evening hyperphagia, mood/sleep, and nocturnal ingestions. The frequencies of responses to NEQ items in our sample are provided in Supplementary Table 1. Two different cut-offs of NEQ total score have been previously proposed for screening purposes (i.e., total score ≥ 25 and ≥ 30) [22]. Since in our sample only two patients showed a total score exceeding the lower cut-off (2 of 121, 1.7%), a different procedure was defined prior to statistical analyses and applied to identify patients with night-eating behavior (NEB) based on core manifestations of night-eating syndrome: (1) evening hyperphagia, or (2) awakenings accompanied by nocturnal ingestions, or (3) both [3, 4]. Evening hyperphagia was defined according to the self-reported percentage of food daily intake after suppertime exceeding 25% (NEQ item 5 score ≥ 2). Nocturnal ingestions were defined based on the presence of weekly night-time awakenings associated with eating or snacking according to NEQ responses (item 9 score ≥ 2 and item 10/11/12 score ≥ 1) (Table 1). Both item 5 and item 9 have been previously used for screening purposes by other authors [26, 27] and have showed high precision in discriminating those with night-eating problems according to item response theory analysis [28]. While for evening hyperphagia (item 5) the usual cut-off was applied, the nocturnal ingestions were defined based on the combination of weekly night awakenings with eating behaviors (items 10 and 12) listed in nocturnal ingestions subscale or with the belief that one must eat in order to get to sleep (item 11), that has been previously deemed critical in the diagnosis of night-eating syndrome [29].

**Table 1:** Criteria used to identify patients with night-eating behavior (N = 20)

Criteria	Description	*N* (%)
Evening hyperphagia	Consume > 25% daily intake after suppertime (item 5 ≥ 2)	7 (35%)
Nocturnal ingestions	Get up at least once a week in the middle of the night (item 9 ≥ 2) *and* craves to eat snacks *or* eats in order to get back to sleep *or* snacks in the middle of the night (items 10/11/12 ≥ 1)	11 (55%)
Both	Evening hyperphagia *and* nocturnal ingestions	2 (10%)

Additional criteria for night-eating syndrome, including morning anorexia and depressed mood or mood worsening in the evening, were not considered in our definition of NEB. On one hand, morning anorexia has been found to scarcely contribute to distinguish night-eaters from other obese patients and its significance as a criterion for night-eating syndrome has been questioned [28]. Mood symptoms were also excluded from criteria used for NEB since this choice could have biased our findings on the associations between night-eating and psychiatric mood comorbidity and based on the lack of discriminant validity in obese candidates to surgery [22]. Moreover, night-eating syndrome criteria used in previous studies have often required the presence of awareness and recall of night-eating episodes, that have been proposed to distinguish night-eating syndrome from sleep-related eating disorders [4, 30]. However, these two conditions are scarcely delimited, often co-occur and show overlapping clinical and video-polysomnographic features [31, 32]. Accordingly, both conditions have been proposed to lie on the same continuum of night-time eating behavior [33, 34], that is presumably captured by our criteria.

The extent of binge-eating and purging behaviors were estimated using the Italian version of the BITE [24], a self-report scale consisting of two subscales: the Symptom Scale (30 items in yes/no format), which measures the degree of symptoms and the Severity Scale (3 items on a Likert scale), which provides an index of the severity based on the frequency of symptoms. The SCL-90-R was used to assess the severity of current general psychopathological symptoms and distress [25]. It is a multidimensional self-rated measure consisting of 90 items rated on a five-point scale from 0 (“Not at All”) to 4 (“Extremely”) specifying how much each symptom has bothered during the past 7 days. Items are assembled into nine symptom dimensions, namely somatization, obsessive–compulsive, interpersonal sensitivity, depression, anxiety, hostility, phobic anxiety, paranoid ideation, and psychoticism. The Global Severity Index was also computed as the average response to all the items to measure general psychological distress.

All the statistical analyses were performed using R Statistical Software (Foundation for Statistical Computing, Vienna, Austria). Shapiro–Wilk test was used for normality check. First, patients with and without NEB were compared using Pearson’s chi-squared tests (or Fisher’s exact test, when needed) for gender and psychiatric comorbidity, Student’s t-test for age, and Wilcoxon rank-sum test for BMI and psychometric scales (including non-definitional NEQ subscales, i.e., morning anorexia and mood/sleep). Based on the hypothesis that significant differences between patients with and without NEB could be, at least in part, attributed to underlying bipolarity, we subdivided patients in four groups according to the presence or absence of both NEB and bipolar spectrum disorders. All the variables showing significant differences between patients with and without NEB in the main analyses were compared among these latter four groups using Fisher’s exact test or Kruskal–Wallis test. Pairwise Fisher’s exact test and Dunn’s test of multiple comparisons were respectively used for post‐hoc comparisons, using Benjamini–Hochberg procedure as false discovery rate (FDR) correction method. Finally, differences in NEQ total and subscales scores among patients without and with different mood disorders (i.e., major depressive disorder, BD type 1 or 2, and cyclothymia or other specified BDs) were investigated by means Kruskal–Wallis test. Dunn's test of multiple comparisons was used for post hoc comparisons, using Benjamini–Hochberg correction method. Supplementary analyses were conducted to test the associations between NEQ total and subscales scores and demographic and clinical variables using Spearman’s rank correlation coefficient for continuous variables, while Wilcoxon rank-sum test was used to look for differences between categories. This latter approach allowed to examine differential correlates of separate night-eating syndrome dimensions. A statistical significance level of *p* < 0.05 was set for all tests.

## Results

Our sample was composed of 121 obese patients referred for bariatric surgery. Most patients were female (*N* = 94, 77.7%). Age ranged between 18 and 66 years (M = 44.31, SD = 11.05). Weight ranged between a minimum of 89 kg to a maximum of 229 kg (M = 127.78, SD = 23.81). BMI ranged between 34.92 kg/m^2^ and 74.70 kg/m^2^ (M = 47.06, SD = 6.86). Most patients were diagnosed with class III obesity (BMI ≥ 40 kg/m^2^), with 105 out of 121 patients affected (86.8%). The other patients were diagnosed with class II obesity (35 kg/m^2^ ≤ BMI < 40 kg/m^2^) except for one who was affected by class I obesity (BMI < 35 kg/m^2^). According to our criteria based on NEQ responses, 20 subjects showed NEB (16.5%). Approximately two thirds of them satisfied the nocturnal ingestions criterion or both criteria for nocturnal ingestions and evening hyperphagia, while about one third only reported evening hyperphagia (Table 1).

Patients with NEB were significantly more frequently diagnosed with bipolar spectrum disorders in comparison with patients without NEB (Table 2). BD type 1 was specifically significantly associated with NEB. Eating disorders were more frequently observed in patients with NEB compared to patients without, however this difference only approached significance (*p* = 0.083). When the comorbidity between eating and mood disorders was considered, a significant association with NEB was observed: eating and mood disorder comorbidity rate in patients with NEB reached more than twice the rate found in patients without NEB. Among non-definitional NEQ subscales only mood/sleep showed a significant difference between groups, while similar scores of morning anorexia were observed in patients with and without NEB. In addition, patients with NEB showed significantly higher Symptom score and Severity score from the BITE. Finally, a significantly higher distress due to somatization, obsessive–compulsive symptoms, phobic anxiety, psychoticism, and sleep disorders was evidenced based on SCL-90-R dimensions in patients with NEB compared to patients without. Correspondingly, patients with NEB scored significantly higher than those without on Global Severity Index.

**Table 2:** Differences between patients with and without night-eating behavior (NEB)

Demographic and anthropometric data	With NEB (*N* = 20)	Without NEB (*N* = 101)	Stats	*p*
	Mean ± SD / N (%)	Mean ± SD / N (%)		
Age (years)	45.6 ± 7.81	44.05 ± 11.61	0.74	0.464
Gender (female)	14 (70%)	80 (79.2%)	0.37	0.542
Body mass index (kg/m2)	48 ± 9.01	46.87 ± 6.39	0.00	0.994
*Psychiatric comorbidity*				
Mood disorders	12 (60%)	43 (42.6%)	1.40	0.236
Major depressive disorder	1 (5%)	14 (13.9%)	–	0.461
Bipolar spectrum disorders	11 (55%)	29 (28.7%)	4.09	**0.043**
Bipolar disorder type 1	3 (15%)	2 (2%)	–	**0.031**
Bipolar disorder type 2	6 (30%)	15 (14.9%)	1.72	0.190
Cyclothymia or other specified BDs	2 (10%)	12 (11.9%)	–	0.999
Panic disorder	5 (25%)	17 (16.8%)	0.30	0.584
Eating disorders	11 (55%)	32 (31.7%)	3.01	0.083
Binge-eating disorder	10 (50%)	31 (30.7%)	1.98	0.159
Bulimia nervosa	2 (10%)	4 (4%)	–	0.258
Eating and mood disorders comorbidity	9 (45%)	19 (18.8%)	5.05	**0.025**
*Night-Eating Questionnaire (NEQ)*				
Morning anorexia	1.6 ± 1.05	1.75 ± 0.93	0.10	0.275
Mood/sleep	4.4 ± 3.02	2.78 ± 2.15	– 0.20	**0.027**
*Bulimic Investigatory Test, Edinburgh (BITE)*				
Symptom score	17.9 ± 7.62	12.31 ± 8.22	– 0.27	**0.004**
Severity score	2.45 ± 2.48	1.36 ± 1.76	– 0.21	**0.024**
*Symptom Checklist—90—Revised (SCL-90-R)*				
Somatization	1.25 ± 0.85	0.73 ± 0.57	– 0.22	**0.016**
Obsessive compulsion	0.75 ± 0.52	0.49 ± 0.63	– 0.23	**0.011**
Interpersonal sensitivity	0.81 ± 0.78	0.57 ± 0.63	– 0.12	0.206
Depression	0.9 ± 0.78	0.56 ± 0.58	– 0.18	0.055
Anxiety	0.76 ± 0.66	0.45 ± 0.48	– 0.16	0.076
Hostility	0.49 ± 0.51	0.34 ± 0.51	– 0.15	0.113
Phobic anxiety	0.44 ± 0.49	0.16 ± 0.38	– 0.23	**0.010**
Paranoid ideation	0.79 ± 0.77	0.52 ± 0.59	– 0.12	0.177
Psychoticism	0.54 ± 0.67	0.24 ± 0.38	– 0.21	**0.020**
Sleep	1.28 ± 1.18	0.64 ± 0.83	– 0.23	**0.011**
Global Severity Index	0.81 ± 0.61	0.49 ± 0.47	– 0.20	**0.027**

Student’s t and Wilcoxon’s r are reported as summary statistics for comparisons of continuous variables, χ^2^ for chi-squared tests. *p* < 0.05 are shown in bold

Differences in eating and mood disorder comorbidity rate, NEQ mood/sleep subscale, BITE Symptom and Severity scores, and SCL-90-R somatization, obsessive compulsion, phobic anxiety, psychoticism, sleep disorders and Global Severity Index were examined among patients subdivided according to the presence or absence of both NEB and bipolar spectrum disorders (Table 3). Eating and mood disorders comorbidity was significantly more frequent in patients with bipolar spectrum disorders, with or without NEB, in comparison with patients without both conditions. Among patients with NEB, those with bipolar spectrum disorders showed significantly more frequently eating and mood disorders comorbidity than NEB patients without bipolar spectrum disorders. Patients with comorbid NEB and bipolar spectrum disorders had significantly higher scores at NEQ mood/sleep subscale than patients without both conditions. Significant differences between these two latter groups were also observed at BITE Symptom score and at SCL-90-R Global Severity Index and obsessive compulsion, phobic anxiety and psychoticism subscales. Global Severity Index, phobic anxiety and psychoticism were also significantly higher in patients with bipolar spectrum disorders without NEB compared to patients without both conditions.

**Table 3:** Differences among patients subdivided according to the presence of both night-eating behavior and bipolar spectrum disorders

	w/o (*N* = 72)	BSDs (*N* = 29)	NEB (*N* = 9)	NEB + BSDs (N = 11)	Χ^2^	*p*	Post hoc contrasts
	Mean ± SD / *N* (%)	Mean ± SD / *N* (%)	Mean ± SD / *N* (%)	Mean ± SD / N (%)			
Eating and mood disorders comorbidity	7 (9.7%)	12 (41.4%)	1 (11.1%)	8 (72.7%)	-	**0.000**	NEB + BSDs, BSDs > w/o NEB + BSDs > NEB
*Night-Eating Questionnaire (NEQ)*							
Mood/sleep	2.53 ± 2.09	3.41 ± 2.21	3.56 ± 2.88	5.09 ± 3.08	9.97	**0.019**	NEB + BSDs > w/o
*Bulimic Investigatory Test, Edinburgh (BITE)*							
Symptom score	11.62 ± 7.88	13.86 ± 8.87	16.33 ± 8.17	19.18 ± 7.28	10.61	**0.014**	NEB + BSDs > w/o
Severity score	1.31 ± 1.82	1.48 ± 1.64	2.67 ± 2.65	2.27 ± 2.45	5.82	0.121	-
*Symptom Checklist—90—Revised (SCL-90-R)*							
Somatization	0.65 ± 0.57	0.91 ± 0.53	1.18 ± 0.91	1.32 ± 0.84	11.35	**0.010**	-
Obsessive compulsion	0.45 ± 0.65	0.59 ± 0.58	0.6 ± 0.56	0.9 ± 0.46	12.06	**0.007**	NEB + BSDs > w/o
Phobic anxiety	0.13 ± 0.39	0.25 ± 0.34	0.48 ± 0.58	0.41 ± 0.41	14.06	**0.003**	NEB + BSDs, BSDs > w/o
Psychoticism	0.18 ± 0.34	0.39 ± 0.42	0.49 ± 0.79	0.6 ± 0.57	15.42	**0.001**	NEB + BSDs, BSDs > w/o
Sleep	0.59 ± 0.79	0.77 ± 0.92	1.3 ± 1.3	1.26 ± 1.13	7.41	0.060	–
Global Severity Index	0.41 ± 0.44	0.67 ± 0.48	0.77 ± 0.71	0.85 ± 0.54	14.94	**0.002**	NEB + BSDs, BSDs > w/o

Kruskal–Wallis’ Χ^2^ are reported. Post hoc contrasts are based on Pairwise Fisher’s exact test and Dunn test with Benjamini–Hochberg correction. *p* < 0.05 are shown in bold. Abbreviations: w/o = without night-eating behavior and bipolar spectrum disorders, *BSDs *bipolar spectrum disorders without night-eating behaviour, *NEB *night-eating behavior without bipolar spectrum disorders, *NEB + BSDs *comorbid night-eating behavior and bipolar spectrum disorders

Finally, subjects were subdivided according to mood disorders in four subgroups: 66 subjects had no major mood disorders, 15 patients satisfied criteria for major depressive disorder, 14 for cyclothymia or other specified BDs and 26 for BD type 1 or 2. Differences in NEQ total and subscales scores were assessed (Table 4). Patients with BD type 1 or 2 scored significantly higher than those without mood disorders in NEQ total score, mood/sleep and nocturnal ingestions subscales. In addition, they also scored significantly higher than patients with either major depressive disorder or other BDs in NEQ nocturnal ingestions.

**Table 4:** Differences in Night-Eating Questionnaire (NEQ) total and subscales scores among patients without and with different mood disorders

	w/o MD (*N* = 66)	MDD (*N* = 15)	CYC (*N* = 14)	BD (*N* = 26)	Χ^2^	*p*	Post hoc contrasts
	Mean ± SD	Mean ± SD	Mean ± SD	Mean ± SD			
NEQ total score	9.30 ± 4.75	10.00 ± 4.69	10.00 ± 2.91	13.88 ± 7.07	9.89	**0.020**	BD > w/o MD
NEQ morning anorexia	1.67 ± 0.95	1.67 ± 1.11	2.21 ± 0.97	1.65 ± 0.8	4.78	0.189	–
NEQ evening hyperphagia	3.65 ± 1.59	3.80 ± 1.66	3.86 ± 1.1	3.81 ± 1.77	0.47	0.926	–
NEQ mood/sleep	2.48 ± 2.03	3.33 ± 2.77	3.36 ± 2.62	4.15 ± 2.52	8.93	**0.030**	BD > w/o MD
NEQ nocturnal ingestions	0.61 ± 2.14	0.33 ± 1.29	0.00 ± 0.00	3.5 ± 5.59	16.13	**0.001**	BD > w/o MD, MDD, CYC

Kruskal–Wallis’ Χ^2^ are reported. Post hoc contrasts are based on Dunn test with Benjamini–Hochberg correction. *p* < 0.05 are shown in bold. Abbreviations: w/o *MD *without mood disorders, *MDD *major depressive disorder, *CYC *cyclothymia or other specified bipolar disorders, *BD *bipolar disorder type 1 or 2

## Discussion

In this study, NEB was evaluated in a sample of 121 obese patients referred for bariatric surgery. To identify all patients presenting with NEB, we decided to use broad diagnostic criteria relying on the core symptoms of the syndrome according to Stunkard et al. [3]. Evening hyperphagia and/or weekly nocturnal ingestions were reported by twenty patients (16.5%). A similar prevalence of NEB or subsyndromal night-eating syndromes has been previously found in patients with class II-III obesity (18.4%) [27] and in pre-bariatric patients (15.1%) [35]. These rates are also consistent with 6–16% prevalence of night-eating syndrome in obese persons and with slightly higher rates found in bariatric surgery candidates [7]. However, since diagnostic tools and operational definitions varied widely in the literature, large differences in prevalence estimates of both NEB and night-eating syndrome have been previously observed.

Indeed, only a small minority of our subjects (1.7%) screened positive at NEQ using a standard cut-off. This prevalence approximates those found in a few samples of bariatric surgery candidates using strict criteria [26, 36]. However, the low prevalence of night-eating syndrome according to NEQ cut-off may have also been due to the tendency of patients to underreport their disordered eating behaviors to access bariatric surgery. No studies are available to quantify the accuracy of reports of patients seeking bariatric surgery compared to patients who completed presurgical evaluation using different assessment methods. Nevertheless, there is some evidence that overweight and obese subjects may underestimate their body weight [37] and underreport their food intake, especially when affected by binge-eating disorder [38, 39]. These findings indirectly support the hypothesis that night-eating could be underreported in pre-bariatric samples and further studies are warranted.

Importantly, several associations between NEB and psychiatric conditions have been observed in our study. As expected, we found higher rates of bipolar spectrum disorders in patients with NEB compared to those without. Although eating disorders were not significantly overrepresented in patients with NEB, mood and eating disorders comorbidity was more frequently observed in this group and patients with NEB also reported a higher severity of binge-eating and purging behaviors according to BITE Symptom and Severity scores. Moreover, these subjects scored significantly higher than those without NEB at SCL-90-R subscales investigating sleep disorders, psychoticism and anxious symptoms, including somatization, obsessive–compulsive and agoraphobic symptoms. Interestingly, several differences between patients with and without NEB could be considered driven by patients with bipolar comorbidity and the associations between night-time eating and bipolar spectrum disorders, mood and eating disorders comorbidity, and bulimic symptoms were confirmed when looking at NEQ nocturnal ingestions subscale correlates (see Supplementary Information).

To the best of our knowledge, this is the first study to identify a significant association between NEB and BD in morbidly obese patients. In the only previous study conducted on night-eating in BD patients, Melo and colleagues [17] found a prevalence of night-eating syndrome almost reaching 10%. Our findings highlight that the prevalence of more broadly defined NEB could be considerably higher in obese BD patients. In our sample, more than one fourth of patients with bipolar spectrum disorders reported NEB (11/40, 27.5%). Increasing rates were observed when moving from broader bipolarity to narrower BD phenotypes. On one hand, NEB was found in only 2 patients out of 14 (14.3%) diagnosed with cyclothymia or other specified BDs; on the other, more than one fourth of patients with BD type 1 (3/5, 60%) or type 2 (6/21, 28.6%) were identified as night-eaters. When subdividing patients according to mood disorders, those with BD type 1 or 2 scored higher on nocturnal ingestions than patients with major depression, with cyclothymia or other specified BD and without mood disorders. Overall, more than a half of patients reporting NEB in our sample were diagnosed with bipolar spectrum disorders (11/20, 55%) and almost all NEB patients diagnosed with mood disorders were affected by bipolar spectrum disorders (11/12, 91.7%). Based on these results, night-eating could be considered indicative of bipolarity in obese patients with mood disorders. Interestingly, among the two patients with positive screening at NEQ according to the standard cut-off, one was diagnosed with BD type 1, with comorbid panic and binge-eating disorder, while the latter failed to show any psychiatric comorbidity.

Several features of night-eating and BD could explain, at least in part, their association. As previously stated, both night-eating syndrome and BD are characterized by circadian disturbances, changes in the timing and amplitude of melatonin secretion and evening chronotype [13, 15, 40, 41]. In addition, diurnal mood variation, possibly associated with bipolarity in depressive patients [42], has been repeatedly observed in night-eaters to the point that it has been proposed as an optional criterion for night-eating syndrome [43]. More importantly, seasonal mood and eating pattern variations have been recently reported in subjects with night-eating and a significant association with seasonal affective disorders has been unravelled [40]. BD is frequently characterized by a seasonal pattern and greater seasonal fluctuations in mood and behavior have been found in individuals with BD than in unipolar depressive patients and controls [44]. Interestingly, comorbid eating disorders [45] and overweight or obesity [46] are more common in patients with BD which show a seasonal pattern of episodes.

Other parallels between night-eating and BD could also be made based on temperamental correlates. Indeed, elevated novelty seeking and increased reward sensitivity have been reported to possibly characterize patients with sleep-related eating disorders that would satisfy our criteria for NEB [47]. These commonalities may be tentatively interpreted according to a recently proposed integrated model of BD linking reward sensitivity to social/circadian rhythm disruption [48]. According to this model, hypersensitivity to reward may lead to states of excessive activation (or deactivation) involving changes in appetitive motivation and response initiation, which in turn are likely to disrupt circadian rhythms and to trigger mood symptoms. On the other hand, circadian rhythm disruption can affect levels of reward sensitivity and activation by timing information generated by the suprachiasmatic nucleus, which is known to interact with brain regions fundamental to the reward system [48]. Within this framework, we posit that evening hyperphagia and nocturnal ingestions may represent an expression of social/circadian rhythm disruption strongly related to changes in appetitive behavior underpinned by the constitutional reward hypersensitivity observed in BD. Importantly, a genetic link between BD and night-eating syndrome has been proposed based on a report of seven cases of night-eating syndrome descending from a couple of subjects diagnosed with BD and delusional disorder [49].

## Strength and limitations

Our study is the first to unravel a significant and specific association between night-eating and bipolar spectrum disorders in severely obese patients. However, some important limitations should be considered. First, the detection of NEB was based on a self-report questionnaire rather than on structured diagnostic interviews, which limits the validity of the results. The recruitment occurred during routinely performed pre-bariatric evaluations, which could lead patients to underreport some aspects of their eating habits to access surgery. Importantly, our NEB criteria did not correspond to some of the previously proposed definitions of night-eating syndrome. Nevertheless, they mostly overlapped with DSM-5 formulation and, in the absence of a valid demarcation between night-eating syndrome and sleep-related eating disorders [33, 34], were aimed to capture the whole spectrum of night-time eating behavior. Finally, the prevalence of current disorders or psychopharmacotherapy were not systematically assessed, and the cross-sectional study design mostly limited the assessment of lifetime psychiatric comorbidity to retrospective accounts, which may be at risk of recall bias. In future studies, it would be interesting to investigate whether night-eating behaviors mainly occur during depressive or (hypo)manic episodes and subside with remission or actually represent a trait-like condition associated with BD.

## Conclusions

There are limited published data about psychopathology related to night-eating. In our study, we observed a strong relationship between bipolar spectrum disorders and NEB. Given that night-eating seems to yield a reduction in weight loss during supervised diets and more complications during weight-loss attempts [50], a better characterization of night-eating psychopathology and psychiatric comorbidity in candidates for bariatric surgery could have clinical and therapeutic implications. Patients with NEB should be carefully evaluated to detect possible mood disorders belonging to the bipolar spectrum. These diagnostic investigations should be considered essential before making any treatment decisions. In case of comorbidity between NEB and BD, the benefits putatively associated with the use of serotonin-selective reuptake inhibitors in the treatment of night-eating syndrome [51] should be balanced with risks associated with antidepressant treatment in patients with BD [52]. In the future, more studies are needed to determine the effect of the association between NEB and BD on short- and long-term outcomes of weight-loss therapies and particularly bariatric surgery.

## What is already known on this subject?

Night-eating is relatively common in obese patients and is often found in comorbidity with psychiatric disorders, such as mood, eating, anxiety, sleep and substance use disorders. Despite shared characteristics, e.g., sleep and circadian rhythms disturbances, the association between night-eating and bipolar spectrum disorders has been under-investigated.

## What this study adds?

To the best of our knowledge, this is the first study to identify a significant association between night-eating and bipolar disorder in morbidly obese patients. Several differences between patients with and without night-eating behaviors are possibly driven by underlying bipolar disorders. Night-eating could be considered indicative of bipolarity in obese patients with mood disorders.

## Supplementary Information

Below is the link to the electronic supplementary material.

Supplementary file1 (DOCX 34 KB)

## Data Availability

Data are available on reasonable request from the corresponding author.
