# Supplementary material for: Prevalence and psychiatric comorbidities of night-eating behavior in obese bariatric patients: preliminary evidence for a connection between night-eating and bipolar spectrum disorders
Source: Eat Weight Disord. 2021 Oct 6;27(5):1695–704. doi: 10.1007/s40519-021-01306-1 (PMC9122845; doi:10.1007/s40519-021-01306-1)
Supplement: Supplementary file 1 — Supplementary file1 (DOCX 34 KB) [file 40519_2021_1306_MOESM1_ESM.docx]

**Prevalence and psychiatric comorbidities of night eating** **behaviour in obese bariatric patients: preliminary evidence for a connection between night eating and bipolar spectrum disorders**

Giulio Emilio Brancati^a^, Margherita Barbuti^a^, Alba Calderone^b^, Paola Fierabracci^b^, Guido Salvetti^b^, Francesco Weiss^a^, Ferruccio Santini^b^, Giulio Perugi^a^

***Supplementary Information***

**Supplementary results**

The associations between NEQ total and subscales scores and other demographic and clinical variables were tested according to a dimensional approach (Supplementary Table 2). With respect to unaffected subjects, NEQ total score was found to be significantly higher in patients with mood disorders (11.84 ± 5.89 vs. 9.30 ± 4.75), bipolar spectrum disorders (12.53 ± 6.19 vs. 9.43 ± 4.71), BD type 2 (13.14 ± 5.86 vs. 9.89 ± 5.18), panic disorder (13.32 ± 7.19 vs. 9.82 ± 4.76), eating disorders (12.23 ± 6.32 vs. 9.47 ± 4.61) and binge-eating disorder (12.24 ± 6.29 vs. 9.54 ± 4.71). The greatest difference was observed between patients with both eating and mood disorders and other patients (13.5 ± 6.76 vs. 9.54 ± 4.61). In addition, NEQ total score significantly positively correlated with BITE Symptom score and the SCL-90-R scores, including all subscales and Global Severity Index.

A similar pattern of associations was found for NEQ mood/sleep subscale. Indeed, higher scores were obtained by patients with mood disorders (3.73 ± 2.60 vs. 2.48 ± 2.03), bipolar spectrum disorders (3.88 ± 2.55 vs. 2.64 ± 2.19), BD type 2 (4.19 ± 2.62 vs. 2.81 ± 2.27) and panic disorder (4.32 ± 3.03 vs. 2.77 ± 2.13) with respect to unaffected subjects. While no significant differences in NEQ mood/sleep subscale were found between patients with and without eating disorders, subjects with both mood and eating disorders scored significantly higher than the others (4.21 ± 2.86 vs. 2.70 ± 2.11). Similarly to NEQ total score, also mood/sleep subscale significantly positively correlated with BITE Symptom score and all the SCL-90-R scores.

Fewer significant associations were demonstrated for NEQ nocturnal ingestions subscale. Among psychiatric comorbidities, only patients with bipolar spectrum disorders (2.27 ± 4.79 vs. 0.56 ± 2.01), more specifically with BD type 1 (8.20 ± 8.14 vs. 0.82 ± 2.59) and, less pronouncedly, type 2 (2.38 ± 4.36 vs. 0.86 ± 2.97), showed significantly higher scores than unaffected subjects. Even in this case, a significant difference was observed between patients with both eating and mood disorders and other patients (2.79 ± 5.42 vs. 0.63 ± 2.09). Moreover, nocturnal ingestions subscale significantly positively correlated with both BITE Symptom and Severity scores, and with SCL-90-R sleep disorders subscale.

As for the other subscales, no significant associations between NEQ evening hyperphagia subscale and other variables were evidenced, while NEQ morning anorexia subscale was found to be lower at increasing ages and higher in patients with cyclothymia or other specified BDs compared to unaffected subjects (2.21 ± 0.97 vs. 1.66 ± 0.93).

**Supplementary Table 1. Frequency of NEQ responses (N = 121).**

|  | 1. How hungry are you usually in the morning? | | | | |
| --- | --- | --- | --- | --- | --- |
|  | *Not at all* | *A little* | *Somewhat* | *Moderately* | *Very* |
| n (%) | 2 (1.7%) | 12 (9.9%) | 12 (9.9%) | 75 (62.0%) | 20 (16.5%) |
|  | **2. When do you usually eat for the first time?** | | | | |
|  | *Before 9 AM* | *9:01 to 12 PM* | *12:01 to 3 PM* | *3:01 to 6 PM* | *6:01 or later* |
| n (%) | 72 (59.5%) | 34 (28.1%) | 13 (10.7%) | 2 (1.7%) | 0 (0%) |
|  | **3. Do you have cravings or urges to eat snacks after supper, but before bedtime?** | | | | |
|  | *Not at all* | *A little* | *Somewhat* | *Very much so* | *Extremely so* |
| n (%) | 59 (48.8%) | 41 (33.9%) | 7 (5.8%) | 13 (10.7%) | 1 (0.8%) |
|  | **4. How much control do you have over your eating between supper and bedtime?** | | | | |
|  | *None at all* | *A little* | *Some* | *Very much* | *Complete* |
| n (%) | 38 (31.4%) | 25 (20.7%) | 18 (14.9%) | 25 (20.7%) | 15 (12.4%) |
|  | **5. How much of your daily food intake do you consume after suppertime?** | | | | |
|  | *0% (none)* | *1–25% (up to a quarter)* | *26–50% (about half)* | *51–75% (more than half)* | *76–100% (almost all)* |
| n (%) | 68 (56.2%) | 44 (36.4%) | 6 (5.0%) | 3 (2.5%) | 0 (0%) |
|  | **6. Are you currently feeling blue or down in the dumps?** | | | | |
|  | *Not at all* | *A little* | *Somewhat* | *Very much so* | *Extremely so* |
| n (%) | 57 (47.1%) | 41 (33.9%) | 12 (9.9%) | 7 (5.8%) | 4 (3.3%) |
|  | **7. When you are feeling blue, is your mood lower in the:** | | | | |
|  | *Early morning* | *Late morning* | *Afternoon* | *Early evening* | *Late evening/nighttime* |
| n (%) | 64 (52.9%) | 3 (2.5%) | 25 (20.7%) | 14 (11.6%) | 15 (12.4%) |
|  | **8. How often do you have trouble getting to sleep?** | | | | |
|  | *Never* | *Sometimes* | *About half the time* | *Usually* | *Always* |
| n (%) | 42 (34.7%) | 57 (47.1%) | 12 (9.9%) | 9 (7.4%) | 1 (0.8%) |
|  | **9. Other than only to use the bathroom, how often do you get up at least once in the middle of the night?** | | | | |
|  | *Never* | *Less than once a week* | *About once a week* | *More than once a week* | *Every night* |
| n (%) | 68 (56.2%) | 20 (16.5%) | 6 (5.0%) | 8 (6.6%) | 19 (15.7%) |
|  | **10. Do you have cravings or urges to eat snacks when you wake up at night?** | | | | |
|  | *Not at all* | *A little* | *Somewhat* | *Very much so* | *Extremely so* |
| n (%) | 38 (31.4%) | 12 (9.9%) | 0 (0%) | 1 (0.8%) | 2 (1.7%) |
|  | **11. Do you need to eat in order to get back to sleep when you awake at night?** | | | | |
|  | *Not at all* | *A little* | *Somewhat* | *Very much so* | *Extremely so* |
| n (%) | 41 (33.9%) | 8 (6.6%) | 1 (0.8%) | 1 (0.8%) | 2 (1.7%) |
|  | **12. When you get up in the middle of the night, how often do you snack?** | | | | |
|  | *Never* | *Sometimes* | *About half the time* | *Usually* | *Always* |
| n (%) | 38 (31.4%) | 13 (10.7%) | 0 (0%) | 0 (0%) | 2 (1.7%) |
|  | **13. When you snack in the middle of the night, how aware are you of your eating?** | | | | |
|  | *Not at all* | *A little* | *Somewhat* | *Very much so* | *Completely* |
| n (%) | 3 (2.5%) | 3 (2.5%) | 2 (1.7%) | 2 (1.7%) | 7 (5.8%) |
|  | **14. How much control do you have over your eating while you are up at night?** | | | | |
|  | *None at all* | *A little* | *Some* | *Very much* | *Complete* |
| n (%) | 5 (4.1%) | 5 (4.1%) | 0 (0%) | 4 (3.3%) | 1 (0.8%) |

**Supplementary Table 2.** **Association between demographic and clinical variables and Night Eating Questionnaire (NEQ) total and subscales scores.** Spearman’s r and Wilcoxon’s r are reported, respectively, for associations with continous and categorical variables. p < 0.05 are shown in bold.

|  | NEQ total score | | NEQ morning anorexia | | NEQ evening hyperphagia | | NEQ mood/sleep | | NEQ nocturnal ingestions | |
| --- | --- | --- | --- | --- | --- | --- | --- | --- | --- | --- |
| Demographic and anthropometric data | **r** | **p** | **r** | **p** | **r** | **p** | **r** | **p** | **r** | **p** |
| Age (years) | -0.05 | 0.680 | -0.30 | **0.003** | 0.06 | 0.616 | -0.05 | 0.686 | -0.10 | 0.374 |
| Gender (female) | -0.07 | 0.441 | -0.06 | 0.479 | -0.08 | 0.353 | -0.12 | 0.180 | 0.09 | 0.304 |
| Body mass index (kg/m2) | -0.03 | 0.792 | -0.12 | 0.264 | -0.01 | 0.958 | -0.11 | 0.307 | 0.09 | 0.407 |
| Psychiatric comorbidity |  |  |  |  |  |  |  |  |  |  |
| Mood disorders | 0.22 | **0.016** | 0.06 | 0.508 | 0.04 | 0.668 | 0.24 | **0.008** | 0.16 | 0.078 |
| Major depressive disorder | -0.03 | 0.725 | -0.07 | 0.456 | -0.02 | 0.834 | 0.02 | 0.839 | -0.07 | 0.429 |
| Bipolar spectrum disorders | 0.25 | **0.005** | 0.11 | 0.220 | 0.06 | 0.546 | 0.24 | **0.008** | 0.22 | **0.015** |
| Bipolar disorder type 1 | 0.13 | 0.149 | 0.09 | 0.312 | -0.10 | 0.296 | 0.10 | 0.292 | 0.33 | **0.000** |
| Bipolar disorder type 2 | 0.23 | **0.013** | -0.07 | 0.419 | 0.12 | 0.206 | 0.21 | **0.021** | 0.22 | **0.018** |
| Cyclothymia or other specified BDs | 0.02 | 0.807 | 0.20 | **0.033** | 0.00 | 0.966 | 0.04 | 0.626 | -0.14 | 0.139 |
| Panic disorder | 0.20 | **0.029** | 0.14 | 0.118 | 0.04 | 0.677 | 0.21 | **0.024** | 0.09 | 0.304 |
| Eating disorders | 0.24 | **0.010** | 0.15 | 0.101 | 0.03 | 0.773 | 0.09 | 0.317 | 0.15 | 0.105 |
| Binge-eating disorder | 0.24 | **0.009** | 0.16 | 0.086 | 0.08 | 0.365 | 0.09 | 0.341 | 0.11 | 0.247 |
| Bulimia nervosa | 0.06 | 0.514 | 0.10 | 0.291 | -0.09 | 0.304 | 0.07 | 0.458 | 0.04 | 0.677 |
| Eating and mood disorders | 0.27 | **0.003** | 0.08 | 0.359 | 0.01 | 0.906 | 0.23 | **0.012** | 0.22 | **0.015** |
| Bulimic Investigatory Test, Edinburgh (BITE) |  |  |  |  |  |  |  |  |  |  |
| Symptom score | 0.36 | **0.000** | 0.18 | 0.120 | 0.07 | 0.590 | 0.23 | **0.035** | 0.33 | **0.001** |
| Severity score | 0.19 | 0.094 | 0.00 | 0.997 | 0.01 | 0.958 | 0.01 | 0.909 | 0.33 | **0.001** |
| Symptom Checklist – 90 – Revised (SCL-90-R) |  |  |  |  |  |  |  |  |  |  |
| Somatization | 0.38 | **0.000** | 0.13 | 0.249 | 0.18 | 0.123 | 0.43 | **0.000** | 0.18 | 0.120 |
| Obsessive compulsion | 0.43 | **0.000** | 0.19 | 0.098 | 0.05 | 0.668 | 0.47 | **0.000** | 0.18 | 0.117 |
| Interpersonal sensitivity | 0.33 | **0.001** | 0.19 | 0.093 | 0.08 | 0.463 | 0.49 | **0.000** | 0.10 | 0.392 |
| Depression | 0.38 | **0.000** | 0.04 | 0.728 | 0.09 | 0.410 | 0.52 | **0.000** | 0.15 | 0.190 |
| Anxiety | 0.41 | **0.000** | 0.15 | 0.188 | 0.12 | 0.288 | 0.48 | **0.000** | 0.16 | 0.155 |
| Hostility | 0.35 | **0.001** | 0.19 | 0.098 | 0.15 | 0.180 | 0.41 | **0.000** | 0.04 | 0.714 |
| Phobic anxiety | 0.28 | **0.007** | 0.05 | 0.678 | 0.04 | 0.744 | 0.34 | **0.001** | 0.16 | 0.161 |
| Paranoid ideation | 0.38 | **0.000** | 0.17 | 0.140 | 0.12 | 0.290 | 0.50 | **0.000** | 0.15 | 0.180 |
| Psychoticism | 0.36 | **0.000** | 0.15 | 0.180 | 0.09 | 0.407 | 0.45 | **0.000** | 0.20 | 0.093 |
| Sleep | 0.41 | **0.000** | 0.06 | 0.630 | 0.12 | 0.307 | 0.32 | **0.001** | 0.22 | **0.049** |
| Global Severity Index | 0.46 | **0.000** | 0.16 | 0.164 | 0.14 | 0.239 | 0.55 | **0.000** | 0.17 | 0.130 |
